# Impact of ubiquitous inhibitors on the GUS gene reporter system: evidence from the model plants Arabidopsis, tobacco and rice and correction methods for quantitative assays of transgenic and endogenous GUS

**DOI:** 10.1186/1746-4811-5-19

**Published:** 2009-12-30

**Authors:** Simone Fior, Paolo D Gerola

**Affiliations:** 1 Department of Structural and Functional Biology, University of Insubria, Via H. Dunant 3, 21100 Varese - Italy

## Abstract

**Background:**

The β-glucuronidase (GUS) gene reporter system is one of the most effective and employed techniques in the study of gene regulation in plant molecular biology. Improving protocols for GUS assays have rendered the original method described by Jefferson amenable to various requirements and conditions, but the serious limitation caused by inhibitors of the enzyme activity in plant tissues has thus far been underestimated.

**Results:**

We report that inhibitors of GUS activity are ubiquitous in organ tissues of Arabidopsis, tobacco and rice, and significantly bias quantitative assessment of GUS activity in plant transformation experiments. Combined with previous literature reports on non-model species, our findings suggest that inhibitors may be common components of plant cells, with variable affinity towards the *E. coli *enzyme. The reduced inhibitory capacity towards the plant endogenous GUS discredits the hypothesis of a regulatory role of these compounds in plant cells, and their effect on the bacterial enzyme is better interpreted as a side effect due to their interaction with GUS during the assay. This is likely to have a bearing also on histochemical analyses, leading to inaccurate evaluations of GUS expression.

**Conclusions:**

In order to achieve reliable results, inhibitor activity should be routinely tested during quantitative GUS assays. Two separate methods to correct the measured activity of the transgenic and endogenous GUS are presented.

## Background

The *Escherichia coli uidA *gene encoding β-glucuronidase (GUS) is one of the most effective reporter gene systems used for evaluating transient and stable transformation in plants. Since its description by Jefferson [1], the GUS gene fusion system has found extensive application in plant gene expression studies because of the enzyme stability and the high sensitivity and suitability of the assay to detection by fluorometric, spectrophotometric, or histochemical techniques. Further advantages lye in the straightforward approach of the GUS assays that do not require expensive equipment, and in the variety of substrates commercially available.

The GUS protein is a 68 kDa homo-tetramer that catalyzes the hydrolysis of β-glucuronides. In most eukaryotic organisms, these are formed to detoxify and excrete xenobiotic and endogenous waste products [1]; in humans, their cleavage by intestinal GUS is known to promote recirculation of toxic compounds responsible for increasing formation of carcinogens (e.g. [2,3]). Increasing efforts are focusing on the study of the role of endogenous GUS in plants, which has been suggested to participate in cell-wall dynamics [4], as well as in the metabolism of secondary compounds, such as flavonoids [5-7]. According to Sudan et al. [4], such endogenous activity should not be considered as a critical limitation to the use of the *E. coli uidA *as a reporter gene, owing to the different pH optima of the two enzymes (i.e. pH 4 and 7, respectively).

Following this wide applicability, it was expectable that shortcomings of the assay would be encountered, and improving protocols developed to render the method amenable to various requirements and conditions (e.g. [8,9]). Unexpected or biased results are common in the use of reporter genes systems, and a vast literature exists with troubleshooting protocols helping solve problems especially for GUS histochemical assays. On the other hand, the GUS reporter gene is often used to quantify gene expression levels within a tissue by extraction of the soluble protein and measurement of GUS activity in the extract with a colorimetric/fluorescent in vitro assay. The fluorometric method described by Jefferson [1] with further implementations (e.g. [10,11]) is widely used to assess promoter activities and compare gene expression patterns from which to infer hypotheses on gene function and regulation. However, some plant extracts may contain components that interfere with GUS activity assay. Thus far, evidence of strong inhibitors of GUS activity has been produced in what seem to be rare cases mostly focused on non-model plants [12-15], thus scarcely considered in the application of the method by the majority of plant scientists. Indeed, the reliability of the quantitative GUS assay has not been addressed in an extensive manner, and artifacts in this method may have been overlooked in the past.

In this paper, we show the ubiquitous presence of inhibitors of *E. coli *GUS activity in the model plants Arabidopsis, tobacco and rice, which create confounding artifacts in the quantitative measurement of GUS activity and are potentially misleading in generating hypotheses on gene studies. Significant levels of inhibitory activity are reported also for plant endogenous GUS, although this is less extensive with respect to *E. coli *GUS. We propose a simple and straightforward method that allows for correction of inhibitor-induced artefacts and we suggest that the inhibitory capacity of the extracts should be routinely tested when performing GUS assays.

## Methods

### Plant material

Leaves, stem in secondary growth, styles and pollen of *Nicotiana tabacum *var. Samsun were collected from one-year old flowering plants grown in controlled environmental conditions under a 12-hour photoperiod at 22/18°C day/night temperature. Light was provided by 400 W Philips HDK/400 lamps. Since growth conditions may alter the content in secondary compounds of plant tissues, the level of inhibitory activity in the above-mentioned organs was also tested in flowering plants grown in the garden. Stem in primary growth and roots were collected from one-month old plants germinated from sterilized seeds sown on soil.

Rosette leaves, inflorescence stems, flowers and fully-developed siliques of *Arabidopsis thaliana *ecotype Columbia were collected from adult (four- to six-weeks old) plants originated from sterilized seeds sown on soil. Plants were grown in the same controlled environmental conditions as *N. tabacum*. Roots were collected from plants grown *in-vitro *on MS medium two weeks after sowing.

Adult leaves of *Oryza sativa *var. Nipponbare were collected from six-weeks old plants originated from sterilized seeds sown on soil. Pants were grown at 25°C under a 12-hour light period. Roots were collected from seedlings obtained from sterilized seeds germinated in Petri dishes on Whatman paper soaked in water.

For experiments based on transgenic GUS (T-GUS), we used transformed plants of *A. thaliana *and *N. alata *containing the *E. coli uidA *gene. This was driven by the constitutive promoter CaMV 35S in *A. thaliana *(gently gifted by Morandini P., University of Milan), and by the pollen specific LAT52 promoter in *N. alata *[16]. Pollen of transformed *N. alata *was used to pollinate pistils of *N. tabacum*. Flowers were emasculated 2 days before anthesis, and covered by a gauze layer. At anthesis, the stigma was covered by a drop of maize oil and pollinated by contact with a mature anther collected from transformed plants of *N. alata *[17]. Styles were collected 48 hours after pollination. As revealed by aniline-blue staining [16], at this time pollen tubes reached the lower part of the style.

### Tissue extraction

Plant tissue was homogenized in ice using a TissueRuptor (Qiagen, Valencia, USA) in 10 mM EDTA, 5.6 mM β-mercaptoethanol and 0,1 M appropriate buffer: sodium phosphate adjusted at pH 7 was used for all experiments except for assays performed on plant endogenous GUS where sodium citrate adjusted at pH 4 was used (see [4]). In all experiments unless otherwise specified, extracts were prepared at a concentration of 100 mg of fresh tissue/ml of extraction buffer. The homogenate was centrifuged at 15000 × g for 20 min at 4°C and the supernatant collected. To avoid artefacts due to the plastic material employed in the experiments, Eppendorf AG (Hamburg, Germany) bio-pure tips and tubes were used (see [11]). To test for possible flaws in the extraction procedure that could bias the measured enzymatic activity, homogenization was also performed by grinding the plant tissue in liquid nitrogen, and in the presence or absence of insoluble PVP (10% w/w; [14]). Consistent results were obtained independently of the extraction procedure employed.

### Fluorometric assay of GUS activity

GUS activity was measured by fluorometric continuous monitoring as described by Fior et al. [11] for most experiments. Repetition of some of the assays using the standard procedure of discontinuous measurement [1] confirmed the consistency of the two methods as previously reported [11]. In particular, while continuous monitoring is a faster procedure, discontinuous measurement has proved a convenient alternative in case of either extremely high or low reaction rates, as it allows for high dilutions of the reaction product and multiple long-time assays.

Assays were performed at 20°C in the extraction buffer with 1 mM 4-methyl-umbelliferyl-β-D-glucuronide (MUG; Sigma-Aldrich Co., Germany) as the substrate. The use of the extraction buffer as reaction medium allowed to measure GUS activity in the presence of any amount of plant extract and therefore to limit the dilution of the extract when the highest enzyme/inhibitor concentration was desired.

To test the inhibitory activity of plant extracts against *E. coli *GUS, the commercial enzyme (Sigma-Aldrich Co., Germany; type VIIA, ca. 1000 units/vial) was employed. The lyophilized enzyme was suspended in 1 ml of water/vial to produce a stock solution, and mother solutions were freshly made by further 1:10 dilutions in the extraction buffer. The inhibition of *E. coli *GUS by plant extract components was measured by comparing the bacterial enzyme activity as measured at the same concentration in the extraction buffer (i.e., the blank sample) and in the plant extract at the convenient dilutions.

The quantification of commercial *E. coli *GUS activity in the presence of plant extract was carried out in two steps. First, MUG was added to the reaction medium containing the plant extract as conveniently diluted (30 μl of 10 mM MUG to 270 μl of reaction medium), and the reaction assayed. This enabled to measure any T-GUS/endogenous activity in the plant extract, when present. When required, maximum enzyme/inhibitor concentration was retained by addition of the substrate directly to the crude extract. Successively, the commercial GUS was added to the assay (10 μl of GUS mother solution to the 300 μl assay solution), and the total enzymatic activity re-assessed. The reaction rates of the commercial GUS were deduced by subtracting any T-GUS/endogenous activity assessed in the previous step from the final enzymatic activity. Following this procedure allowed to assess the inhibitory activity directly in the plant extract where the GUS activity was being measured. This approach has been used in all the experiments carried out in this work.

Continuous monitoring was performed in a thermostatable spectrofluorometer by exciting at 360 nm and following the increase in fluorescence at 480 nm (see [11]). For discontinuous measurement, we followed the protocol described by Jefferson [1]: MU production was measured fluorometrically at 365/453 nm excitation/emission wavelengths.

Fluorescence measurements were performed in a Jasco F-850 spectrofluorometer with a 150 W xenon exciting lamp. In order to convert the measured Δ fluorescence into moles of MU produced during the reaction, both methods required correction from MU fluorescence quenching caused by interference with components present in the plant extract, as described by Fior et al. [11].

### Protein content determination

Protein concentration in the plant extracts was measured by applying the Bradford's method [18] using the Sigma reagent and the bovine serum albumin as a standard.

### Properties of inhibitor molecule

To estimate the molecular weight of putative inhibitor compounds, leaf extracts of the three model plants were fractionated through Centricon filter devices (Millipore, Billerica, USA) following the manufacturer's protocol. Two fractionation steps were performed in series: the plant extract was filtrated through a 30 kDa filter and the flow-through filtrated through a 10 kDa one. The inhibitor activity was compared in the plant extract and in the two filtrates.

## Results

### Assessment of inhibitor activity towards E. coli GUS

The presence of GUS inhibitors was examined in tissue extracts obtained from various organs of the three model plants. The inhibitor activity was calculated by comparing the enzymatic activity of commercial *E. coli *GUS as measured in the extraction buffer and in the plant extract (the latter diluted only by the addition of the substrate and the commercial enzyme, i.e. at 87% concentration. See Materials and Methods for further details on the followed procedure).

All extracts were obtained by grinding 100 mg of fresh tissue/ml of extraction buffer, except for pollen where 10 mg were used. As both the nature and the localization of the inhibitors are currently unknown, it seems arbitrary to relate the inhibitor activity of each organ to the concentration of a particular class of compounds present in the extract, such as, for example, to protein or DNA content. On the other hand, this experiment aims to provide an overall comparison of the inhibitor activity that is present in the organ extract, irrespectively of the types of tissue that constitute the organ. For this reason, the inferred inhibitor activity is referred to the milligrams of fresh weight material ground for each organ.

**Figure 1:**
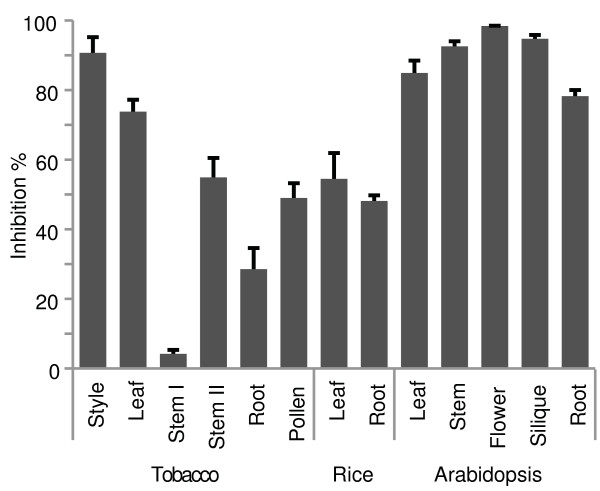
**Percentage of inhibition in different organs of the model plants Arabidopsis, tobacco and rice**. Extracts were obtained from 100 mg/ml of fresh tissue for all organs except for tobacco pollen, where 10 mg/ml were used. Substrate and commercial *E. coli *GUS were added directly to the plant extract in order to minimize extract dilution. Inhibition against commercial *E. coli *GUS was calculated by comparing the measured reaction rate with that assayed in the extraction buffer. Stem I and II refer to stem in primary and secondary growth, respectively. Values are the means ± standard deviation from three independent extractions.

Our results show that significant inhibitory activity is present in all examined organs of the three model plants (Figure 1). Highest inhibitory capacity (> 90%) is associated to the extracts obtained from reproductive parts of Arabidopsis including inflorescence stem, flowers and siliques, as well as from tobacco styles. Strong inhibition (70-85%) is also observed in extracts of leaves and roots of Arabidopsis and in leaves of tobacco, while lower levels (30-60%) are observed in the extracts of rice (both leaves and roots) as well as in those of tobacco roots, pollen and stem in secondary growth. A similar low level of inhibition is recovered after 1:10 dilution of extracts of all Arabidopsis organs (see data reported in the section pertaining inhibition of transgenic *E. coli *GUS), tobacco leaves and styles, implying that a significant effect on the GUS assay can be retained also in highly diluted extracts in case of abundant inhibitor concentration. It is noteworthy that virtually no inhibition was measured in the extracts of tobacco stem in primary growth.

Extraction experiments were repeated thrice for all different organs: consistent values of inhibition were recovered from each trial, as indicated by the statistical analysis (Figure 1). Consistent results were obtained from tobacco plants grown in the greenhouse and in the garden, suggesting that the inhibitor concentration is not related to the growth conditions (data not shown).

### Characteristics of the inhibitor towards E. coli GUS

Type of inhibition was determined in leaf extracts of the three model plants by Cornish-Bowden plot analysis [19]. In such analysis, enzymatic rates are measured at different substrate and inhibitor concentrations, and data are plotted as the ratio between substrate concentration and reaction rate versus the inhibitor concentration. In case of endogenous inhibitor, the absolute concentration of such compound in the extract is clearly unknown; however, being the inhibitor a component of the extract, relative inhibitor concentrations can be extrapolated by normalizing its concentration in the undiluted extract to the unit. This implies that the relative inhibitor concentration coincides with the relative extract concentration.

Leaf extracts were obtained from wild-type plants, and the activity of added *E. coli *GUS was subsequently measured at different substrate and extract concentrations.

**Figure 2:**
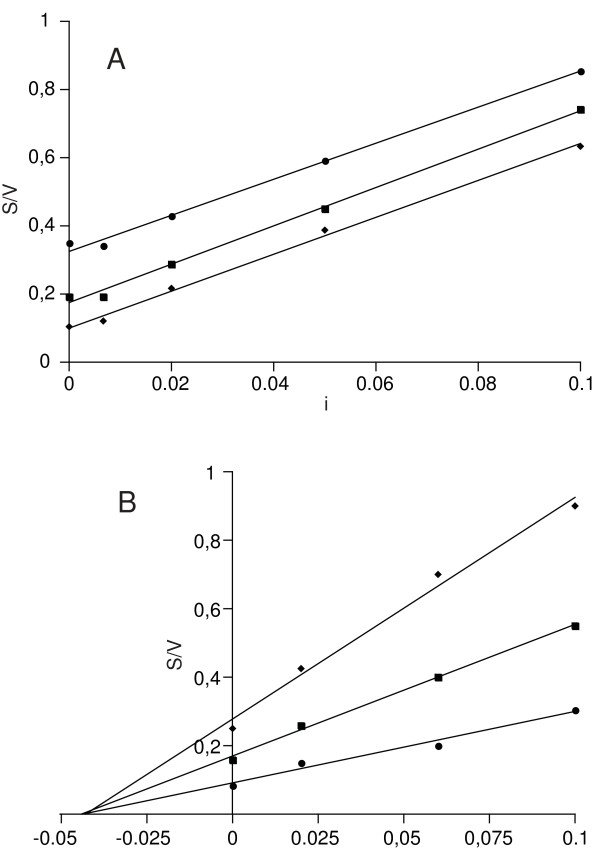
**Type of inhibition in Arabidopsis (A) and tobacco (B) leaf extracts as inferred from the Cornish-Bowden plot**. Enzymatic activity of commercial *E. coli *GUS has been measured in the extraction buffer and in the presence of plant extract at different dilutions (1:10, 1:20, 1:50, 1:150 in A; 1:10, 1:15, 1:50 in B), as well as at different substrate concentrations, i.e., 0.5 (υ), 1 (ν), and 2 (λ) mM MUG for Arabidopsis; and 0.3 (υ), 0.6 (ν), and 1 (λ) mM MUG for tobacco. Reaction rates are were calculated as nanomoles of MU produced per minute per microliter of the commercial GUS mother solution added. S/V indicates the ratio between substrate concentration and reaction rate; i indicates the relative inhibitor concentration present in the sample: it has been obtained by normalizing the inhibitor concentration present in the plant extract at the different dilutions to that in the undiluted extract. It corresponds to the reciprocal of the plant extract dilution factor.

The inhibitor was determined to be competitive in Arabidopsis (Figure 2A), whereas non-competitive inhibition was found in tobacco (Figure 2B) and rice (data not shown).

Preliminary investigations on the nature of the inhibitor were conducted by a rough esteem of its molecular weight. Leaf extracts were filtrated in series through a 30 kDa and a 10 kDa filter device. In all the samples, the inhibitory capacity observed in the plant extract before filtration was completely recovered in the 10 kDa filtrate, indicating a molecular weight of the inhibitors <10 kDa in the three model plants.

### Inhibition of transgenic E. coli GUS

Since post-translational changes associated to GUS synthesis in plants might alter the affinity of the enzyme to extract components, inhibition of the enzyme encoded by the *uidA *transgene (i.e. T-GUS) was assessed and compared to that of the commercial enzyme.

**Figure 3:**
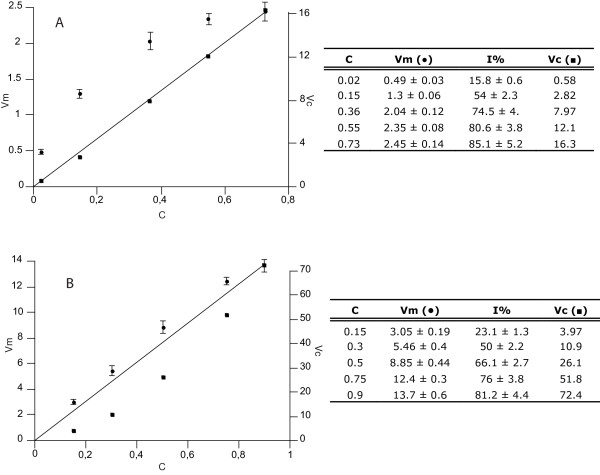
**Activity of transgenic (A) and endogenous (B) GUS in Arabidopsis leaves as measured experimentally and corrected for the estimated inhibitory capacity of the extracts**. Plots are inferred from data reported in the table annexed to each graph. Extracts are obtained from transformed plants in (A), and wild type plants in (B). Vm and Vc indicate the measured and corrected reaction rates, respectively; C indicates the relative extract concentration, calculated by normalizing the undiluted extract to the unit. As the inhibitor and the enzyme are components of the extract, their relative concentrations coincide with the relative extract concentration. Transgenic and endogenous GUS activity have been measured at different extract concentrations: values (Vm; λ) are the means of triplicate measurements ± standard variation. Inhibitory capacity against commercial *E. coli *GUS was assessed for each replicate and is reported as percentage of inhibition (I%) as the mean value ± standard variation (refer to Materials and Methods for the detailed procedure followed to produce the data). The estimated percentage of inhibition against *E. coli *GUS was applied to correct the respective measured reaction rates and the obtained values are reported (Vc; ν). In (A), the trend line interpolates the corrected data, whereas in (B) it simply represents the linearity between extract dilution and activity expected in the absence of inhibitors. Reaction rates are expressed as nanomoles in (A) and picomoles in (B) of MU produced per minute per milligram of protein of the undiluted extract. Left y axis refers to the measured values (Vm), right to the corrected values (Vc).

T-GUS activity was assayed in extracts of transformed leaves of Arabidopsis and of tobacco styles containing transformed pollen tubes. Reaction rate was measured at different extract dilutions and plotted accordingly (Figure 3A). As the plant extract contains both the enzyme and the inhibitor, a linear proportion between enzyme activity and dilution is expected in case of either irreversible inhibition or absence of inhibitor. In case of reversible inhibition, dilution of the extract implies a shift of the enzyme-inhibitor equilibrium towards the undissociated form, resulting in a gain in enzyme activity proportional to the dilution factor and yielding a convex curve in the plot. Such pattern was found for both tobacco (data not shown) and Arabidopsis extracts (Figure 3A), indicating the reversibility of both the non-competitive and competitive inhibition found in these plants.

In order to assess the inhibitory capacity of the extract at each dilution, commercial *E. coli *GUS was added to the transgenic plant extracts at each concentration used in the T-GUS assay. Comparison of the commercial GUS activity measured in the samples with that assayed in the blank allowed the inhibitory capacity to be calculated at each dilution (see Materials and Methods for further details on the followed procedure).

Further, the inhibitory capacities inferred from this experiment (data in Figure 3A) were used to correct the T-GUS reaction rates measured at the respective extract dilutions. When the corrected rates are plotted versus the extract concentration, a direct proportionality is recovered both in the Arabidopsis (Figure 3A) and tobacco (data not shown) graphs. These results demonstrate that the synthesis of the bacterial GUS in transgenic plants does not alter its sensitivity to plant extract inhibitors.

This evidence forms the basis on which to infer a correction method where the inhibition measured on commercial GUS is used to calculate uninhibited reaction rates, and can be applied to all quantitative GUS assays on transformed plants.

### Inhibition of plant endogenous GUS

To test whether extract components affect also plant endogenous GUS, fluorometric assays were performed on leaf extracts of wild type Arabidopsis and tobacco plants. Extracts were obtained by grinding 400 mg of fresh weight material/ml of extraction buffer at pH 4; which is reported as optimal for endogenous GUS activity assay [4]; all the following enzymatic assays were performed in the same buffer.

The endogenous GUS activity present in plant extracts was measured at different extract dilutions and results plotted accordingly (Figure 3B). Similarly to the plot described above, the reaction rates measured at different extract concentrations define a convex curve, though their variation from the expected linearity is considerably smaller as compared to that obtained for the T-GUS (cf. Figure 3A). Since the extraction and assay of endogenous GUS are here performed at pH 4, we tested whether the acidic buffer affects the affinity of the inhibitor towards the *E. coli *enzyme, which could also justify a decreased inhibitory capacity towards the endogenous GUS. In order to test this hypothesis, the inhibitory capacity of the acidic plant extracts against the bacterial enzyme was tested at the different dilutions. Commercial *E. coli *GUS was added to each extract at the different concentrations used in the GUS assay, and its activity compared to that measured in the blank. The obtained results show that the pH does not influence the inhibitor-enzyme interaction, as the inhibitory capacity towards *E. coli *GUS is maintained also at pH 4 (data in Figure 3B). Therefore, the lower inhibitory capacity towards the endogenous GUS is due to a different affinity between the plant enzyme and the inhibitory compounds present in the extract. As a consequence, when the inhibitory capacities assessed against the bacterial enzyme are applied to correct the reaction rates measured for the endogenous GUS (data in Figure 3B), they yield an overestimation of the enzymatic activity. Plotting of the data (Figure 3B) yields a concave curve instead of a straight line as observed in the analogous analysis conducted on T-GUS (cf. Figure 3A).

This evidence rules out the possibility to apply to the endogenous GUS the procedure previously described for the correction of the activity of the transgenic enzyme, and another approach should be followed.

We deduced a simple procedure based on an implementation of the graphical representation of enzyme kinetics as described in the Dixon plot [20], in which the reciprocal of the enzymatic rate measured at different inhibitor concentrations is plotted versus the inhibitor concentration (in our case coinciding with the extract concentration). A straight line is retrieved by interpolating the plotted data, independently from the type of inhibitors present in the extract (competitive, uncompetitive or mixed, which in turn includes non-competitive). The intercept on the y axis (i.e. null inhibitor concentration) corresponds to the reciprocal of the uninhibited enzymatic reaction rate (see Additional File 1. Dixon plot: a graphical method for evaluating the uninhibited reaction rate in a sample in the presence of an enzyme and an inhibitor at unknown concentrations).

**Figure 4:**
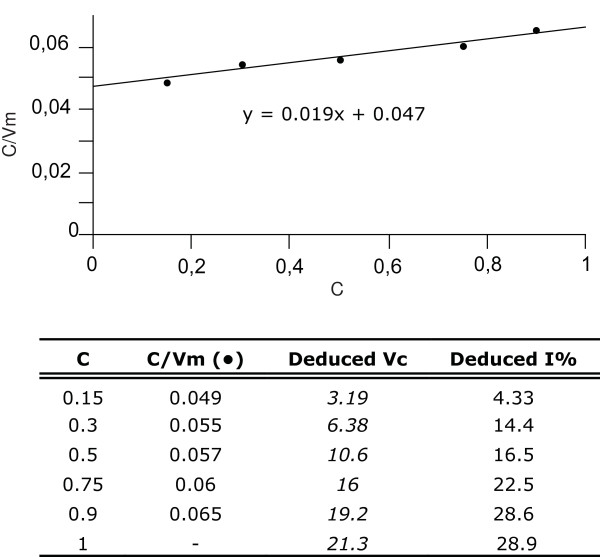
**Dixon plot of endogenous GUS activity measured in Arabidopsis leaf extract**. The plot is inferred from the data reported in the annexed table. These, in turn, are extrapolated from the experimental values reported in figure 3B. C indicates the extract concentration. In order to produce data suitable for the Dixon plot (refer to the Results section and Additional Material for further description), the measured reaction rates were corrected by the dilution factor (i.e. the reciprocal of the extract concentration). The reciprocals of the obtained values (C/Vm) were plotted versus the relative extract concentration, which coincides with the relative inhibitor concentration (As explained in Figure 3B). The intercept of the trend line provides the uninhibited activity in the undiluted extract, thus reported for C = 1 in the table. Uninhibited rates (Vc) at the extract dilutions used in the experiment are deduced by multiplying the latter value by the extract concentration; the resulting values are reported in italics in the table. Finally, the inhibitory capacity (I%) at each dilution can be deduced by comparison between the uninhibited reaction rates and the experimental values (data to Figure 3B), and it is here reported as percentage of inhibition. In this respect, when C = x = 1 (i.e. in the undiluted extract), the inhibited reaction rate corresponds to the reciprocal of the y value in the trend line equation, which provides the value (i.e. 15.2) from which to infer the percentage of inhibition in the undiluted extract (28.9%). Reaction rates are expressed as picomoles of MU produced per minute per milligram of protein of the undiluted extract.

It is important to underline that the Dixon plot applies to assays performed with a constant enzyme concentration whose activity is measured at different inhibitor dilutions; in our case, as the inhibitor is contained in the extract together with the endogenous enzyme, dilution of the inhibitor implies an analogous dilution of the enzyme concentration. This requires that the measured reaction rates are corrected for the dilution factor in order to obtain data suitable for the Dixon plot (see data in Figure 4).

In our results, the Dixon plot yields a slanted trend line and ANOVA of the regression strongly support the evidence that the trend line is not parallel to the x axis, which would indicate total absence of inhibition. Our analyses show that the linear relationship between the two variables is highly significant (b_1 _= 4.65; F_(1,10) _= 43.2; *P *= 6.3 × 10^-5^), with a determination coefficient for b_1 _of R^2 ^= 0.78.

Inhibitory capacity of the undiluted extract was calculated by comparing the inhibited and uninhibited reaction rates of the endogenous enzyme as inferred from the trend line equation of the Dixon plot. More specifically, the inhibited activity is obtained when x = 1, while the uninhibited activity corresponds to the reciprocal of the intercept on the y axis. In our results, the inhibitory capacity corresponds to 29% and 21% in Arabidopsis and tobacco, respectively.

## Discussion

### Impact of plant inhibitors on the GUS system

Since Jefferson's pioneer work [1,21,22], *E. coli *GUS has become one of the most widely used reporter genes in plants. However, analysis of GUS assays is often complex in plant cells, as GUS gene expression is affected by various biochemical, molecular and biological factors that render its use as a reporter gene susceptible to limitations. Improving techniques have been developed especially for histochemical assays, where the GUS gene has been extensively used due to its high sensitivity as visual marker even in a single cell. Protocols have been developed to tackle potential limitations such as background activity (normally due to endogenous enzyme, bacterial contamination, or diffusion of the reaction product), quenching and lethality of the GUS assay, and recently, dual reporter systems have been developed to improve the versatility of the GUS system and compensate for the low turnover rates of the GUS protein (e.g. [23]).

Inhibitors of enzyme activity are a potential cause of significant artifacts both in histological and quantitative assays, but while their effects are difficult to evaluate in the case of colorimetric reaction in plant tissue, extraction of soluble components allows for assessment of inhibitory capacity in GUS fluorometric assays. Presence of inhibitors in tissue extracts interferes in the quantification of transgene expression levels, causing bias in the assessment of promoter activity and leading to generate erroneous hypotheses. The relevance of this applies to all studies based on plant transformation experiments, but obviously acquires a more vast and general interest in the use of model plants.

Our results show that inhibitory capacity is virtually ubiquitous in Arabidopsis, tobacco and rice, and significantly affects quantification of the GUS activity. This evidence combined with previous reports of inhibitors in non-model plants such as cranberry, wheat and carnation plants [12,14,15] suggests that inhibitors of GUS activity may actually be common components of plant cells. Thus far, non-competitive inhibition has been reported for all the above-mentioned plants, as well as for callus extracts of tobacco [24], and the same type of inhibition is here recovered in leaf extracts of tobacco and rice. However, inhibition in leaf extracts of Arabidopsis is determined to be competitive, indicating that inhibitors may vary among plant species and interact differently towards the GUS enzyme. Despite this, inhibition was determined to be reversible in all three model plants.

The reversible nature of the inhibition implies that the inhibitory capacity of the extract decreases following dilution. Thus, GUS assays most prone to artifacts are those performed following transformation with promoters yielding a low concentration of enzyme in the extract, such as, for example, experiments that use weak promoters or promoters active in a limited portion of the sampled tissue, which are usually tested undiluted to allow measurement of the reaction. On the contrary, enzyme activity resulting from strong/constitutive promoters allows high dilution of the extract, in which case the inhibitory capacity may become negligible and the measured activity exempt from artifacts. This finds experimental support in our experience with GUS assays performed on 35S-GUS Arabidopsis leaf extracts: activity of the transgene can be efficiently measured by continuous monitoring even at a 1:1000 dilution of the extracts, where the concentration of the inhibitor is too low to interfere with the reaction (results not shown). Dilution of the extract may also explain the absence of inhibitor reported by previous authors in tobacco leaves [12,14].

Inhibitory capacity may vary among plant organs, yet the small variation found in our results suggests a consistent concentration of inhibitors in each tissue. Thus far, improvements in the extraction procedure have aimed to limit the effects of inhibitors by eliminating secondary compounds through filtration [24] or inactivation with binding reagents (e.g. PVPP; [13]), but complete removal of the inhibitory capacity could not be achieved. In this article we describe a simple and straightforward procedure that allows to correct for enzyme inhibition independently from the level of T-GUS activity present in the plant extract.

Owing to the ubiquitous presence of inhibitors in plant tissues, we strongly suggest that the presence of such compounds interfering in the GUS assay should be tested routinely prior to each GUS assay. This can be easily achieved by measuring the enzymatic rate at two considerably different concentrations of the extract, in order to verify the proportionality between the measured reaction rates and the dilution factor. An enzymatic activity of the least concentrated extract significantly higher than expected clearly indicates the presence of inhibitors which interfere with the measurement.

Hence, in order to obtain reliable assays of T-GUS activity, the inhibitory capacity of the plant extract at the desired concentration should be determined and used to correct the measured enzyme activity.

This is easily achievable because of the same affinity of the plant extract inhibitors towards the enzyme encoded by the *uidA *gene (i.e. T-GUS) and the pure bacterial GUS, which allows to measure the inhibitory capacity of a tissue extract on the commercially available *E. coli *enzyme and apply the correction on the activity of the transgenic enzyme. This involves a straightforward procedure consisting of three steps that can be routinely applied to any assay: a) measurement of the T-GUS activity in the plant extract at convenient dilution; b) addition of a known concentration of commercial *E. coli *GUS to the plant extract and measurement of the overall enzymatic activity; c) measurement of the uninhibited activity of the same concentration of *E. coli *GUS in the extraction buffer. The *E. coli *GUS activity in the plant extract is calculated by subtracting the T-GUS activity from the overall enzymatic activity. The inhibitory capacity is then calculated by comparison of the uninhibited activity of the bacterial enzyme and its activity in the extract, and the inhibition percentage is applied to correct the T-GUS reaction rates. It is important that the same extract concentration (i.e. the inhibitor concentration) is used when T-GUS activity is measured in the presence and absence of the added commercial *E. coli *GUS. Fluorescence coefficient of MU needs to be assessed in order to express enzyme activity in absolute values, thus correcting for fluorescence interference of extract components. This applies regardless of the method employed to assay GUS activity, as we found that extract components can bias results in both continuous and discontinuous measurement (see [11]).

As the nature, localization and regulation of the compounds responsible for GUS inhibition are still unknown, it is difficult to hypothesize what variable factors may affect their concentration and extractability. Moreover, quantification of GUS activity is often performed in experiments carried out under variable physiological conditions, which may bear an unpredictable effect on the presence and activity of the inhibitor. For this reason, assuming that the inhibitory capacity calculated for a certain organ is maintained constant in separate trials may lead to inaccurate results, and we strongly suggest that the inhibitor capacity is calculated for each single extract sample at the desired dilution rather than applying values of correction calculated on separate, independent extracts of the same tissues.

### Inhibition of plant endogenous GUS

Endogenous GUS has recently been shown to be ubiquitous in plants [4], but its characterization is still poorly known. Combined with histochemical evidence coming from a large number of species, quantitative GUS assays performed on model plants have been used to study the activity of the endogenous enzyme (e.g. [4,25-27]), and further investigations will allow to achieve a better understanding of its function in plant organs.

According to our results, inhibitory components present in tissue extracts affect also plant endogenous GUS, although with remarkably lower affinity as compared to the *E. coli *enzyme. Similar disparity of inhibitory capacity towards plant endogenous GUS was previously reported for the commonly used inhibitor of *E. coli *GUS D-saccaric acid 1-4 lactone [24].

As a consequence, the bacterial enzyme cannot be used to correct the activity of the endogenous GUS as described for the transgene. On the other hand, due to the low level of endogenous GUS activity, plant extract dilution can hardly be considered a suitable approach to reduce the inhibitor concentration. We propose that the uninhibited activity of the enzyme in the extract is inferred from an implementation of the analysis of the enzyme kinetics as described in the Dixon plot. This requires that the enzyme activity is measured at different extract dilutions and the results plotted as follows: x axis refers to the extract concentration (i.e. the relative inhibitor concentrations obtained by normalizing the undiluted extract to the unit), while y axis refers to the reciprocal of the reaction rates corrected for the dilution factor. In this plot, the trend line is parallel to the x axis when no inhibition is present in the plant extract. Contrarily, the trend line equation allows to calculate both the uninhibited and inhibited reaction rates in the undiluted extract: the former is yielded by the reciprocal of the intercept on the y axis, the latter by the reciprocal of the y value when x = 1. The inhibitory capacity of the tissue extract can be consequently deduced.

In order to achieve reliable results, we suggest that a suitable number of extract dilutions are measured and we reiterate that the interference of extract components on MU fluorescence must be taken into account in determining the reaction rates (see [11])

To our knowledge, such development of the Dixon equation has never been proposed, yet it allows to assess correct enzyme activity regardless of the availability of pure enzyme and *a priori *of any knowledge of the inhibitory capacity of the solution. It can be employed in any reversible inhibition, independently of its type, and can find general applicability in the measurement of any enzymatic activity.

## Conclusions

GUS inhibitors represent a source of potential artifacts in the use of the *uidA *reporter gene, and the supposed ubiquity of such components in plant tissues urges for careful examination of their interference in GUS expression analyses. The low affinity towards plant endogenous GUS seems to rule out a specific role of inhibitors in the regulation of the plant enzyme, and the simplest explanation of their impact on the bacterial GUS would suggest a casual side effect in the interaction between the enzyme and compounds normally present in plant cells. On the other hand, little evidence is thus far accessible to pinpoint the role of endogenous GUS in plants, and hydrolysis of the fluorogenic substrate has been suggested to occur also by interaction with non-enzymatic compounds present in plant tissues. Such GUS-like activity is likely to be unaffected by GUS inhibitors [24], and may confuse the interpretation of the activity attributable to the endogenous enzyme as well as the determination of the inhibitory capacity.

The method here described provides a fast and efficient tool to produce reliable results that shall find general consensus in the wide use of the GUS system in plant molecular biology, as well as in future studies on the function of plant endogenous GUS. It remains however difficult to ascertain what effects inhibitors may have during the processes of histological GUS assays. It has been suggested that likely inhibitors of GUS activity are non-proteinaceous molecules possibly belonging to the large class of phenolic compounds present in plant cells. The low molecular weight and the ubiquitous presence of inhibitors found in Arabidopsis, tobacco and rice further support this hypothesis. If this holds true, phenolic compounds are most likely sequestered in vacuoles and cell walls, and disruption of cell membranes during the histochemical assay reaction is likely to cause mixing of the inhibitors with the cytoplasmic GUS. Further, the type and amount of phenolic compounds vary widely among plant species, tissues, stages of development and physiological condition, thus the degree of GUS inhibition might be subject to great variation that can account for inaccurate evaluations of GUS expressions.

## Competing interests

The authors declare that they have no competing interests.

## Authors' contributions

SF carried out the experiments, analysed the data and drafted the manuscript. PG conceived of the study, designed and coordinated the experiments, and participated in drafting the manuscript. All authors read and approved the final manuscript.

## Supplementary Material

Additional file 1 Dixon plot: a graphical method for evaluating the uninhibited reaction rate in a sample in the presence of an enzyme and an inhibitor at unknown concentrations Click here for file
