# Supplementary material for: Impact of ubiquitous inhibitors on the GUS gene reporter system: evidence from the model plants Arabidopsis, tobacco and rice and correction methods for quantitative assays of transgenic and endogenous GUS
Source: Plant Methods. 2009 Dec 30;5:19. doi: 10.1186/1746-4811-5-19 (PMC2805642; doi:10.1186/1746-4811-5-19)
Supplement: Additional file 1 — Dixon plot: a graphical method for evaluating the uninhibited reaction rate in a sample in the presence of an enzyme and an inhibitor at unknown concentrations [file 1746-4811-5-19-S1.DOC]

**ADDITIONAL FILE 1**

*Dixon plot: a graphical method for evaluating the uninhibited reaction rate in a sample in the presence of an enzyme and an inhibitor at unknown concentrations*

Dixon plot is generally used as a graphical method for determining enzyme inhibitor constants (Dixon, 1953). Three different formula describe the relationships between the reciprocal of velocity (1/v) and the inhibitor concentration ([I]) in competitive (a) uncompetitive (b) and mixed (c) inhibition:

a)

b)

c)

where $\left[ S \right]$ indicates the substrate concentration, $V\text{max}$the velocity at saturating substrate concentration, the Michaelis-Menten constant, and $K\text{ic}$the dissociation constants of the enzyme-inhibitor complex and of the enzyme-substrate-inhibitor complex, respectively.

The three formula differ for the angular coefficient, and a general equation can be simplified as follows:

where depends on the type of inhibition.

The intercept on the y axis can be formulated as . This value is independent from the type of inhibition and is equivalent to the reciprocal of the uninhibited reaction rate ($\left[ I \right]$=0).

In common experiments investigating inhibitor properties, such value is usually inferred from the experimental data obtained from the control assay with no inhibitor added to the enzyme, but indeed, the intercept of the Dixon plot provides the same information. The significant advantage of this, is that the uninhibited activity of an enzyme in a solution can be inferred correctly without any prior knowledge of either the inhibitor or the enzyme concentration, and indeed, such procedure can be applied to prove the presence/absence of inhibitors and correctly assay enzymatic activity independently from the availability of the pure enzyme.

In general, this applies to most assays performed on samples produced by cell/tissue extraction, where the endogenous enzyme and possible inhibitors are present at concentration unknown to the operator. The procedure simply requires that the enzyme activity is assayed at different extract dilutions. These data provide the activity of the endogenous enzyme as measured at different inhibitor concentrations, as required to construct the Dixon plot. However, a significant variation differentiate such procedure from the standard method as commonly applied, in that diluting the extract implies that assays are performed at different enzyme concentrations, whereas the uninhibited activity is assumed to be constant according to the original method described by Dixon. To correct for such enzyme dilution, measured reaction rates need to be multiplied by the dilution factors, on account of the fact that the enzyme activity is proportional to the enzyme concentration also in the presence of inhibitors.

In the final plot (1/v versus [I]), the inhibitor concentration is expressed as the reciprocal of the dilution factor (i.e. the extract is normalized to the unit), and enzyme activity is reported as the reciprocal of the corrected reaction rates. A trend line parallel to the x axis indicates absence of inhibitor, otherwise the intercept on the y axis yields the uninhibited enzyme activity in the undiluted extract. The inhibitor capacity can be deduced by comparison with the measured activities corrected for the dilution factor. The inhibitor activity in the undiluted extract can be calculated by extrapolating from the equation of the trend line the velocity at the inhibitor concentration equals to the unit, i.e. in the undiluted extract.
